# The provision of compulsory school physical activity: Associations with physical activity, fitness and overweight in childhood and twenty years later

**DOI:** 10.1186/1479-5868-5-14

**Published:** 2008-02-29

**Authors:** Verity Cleland, Terence Dwyer, Leigh Blizzard, Alison Venn

**Affiliations:** 1 Menzies Research Institute, University of Tasmania, Hobart TASMANIA 7000, Australia; 2 Murdoch Children's Research Institute, Royal Children's Hospital, Parkville VICTORIA 3052, Australia

## Abstract

**Background:**

To determine whether the provision of higher levels of compulsory school physical activity is associated with higher physical activity and fitness levels and less overweight in childhood and 20 years later.

**Methods:**

As part of the 1985 Australian Schools Health and Fitness Survey, 109 schools reported how much compulsory physical education (PE) and school sport they provided and were classified as low (<110 and <150 minutes/week for primary and secondary schools, respectively), medium (110–149 and 150–189 minutes/week for primary and secondary schools, respectively) or high (≥150 and ≥190 minutes/week for primary and secondary schools, respectively) compulsory physical activity schools by tertile cutpoints. 6,412 children reported frequency and duration of school (PE and sport) and non-school (commuting and non-organised exercise) physical activity and had height and weight measured; overweight was defined using body mass index (BMI) (m/kg^2^) cutpoints. 9, 12 and 15 year-olds (n = 2,595) completed a cycle ergometer fitness test (physical working capacity at heart rate 170, PWC_170_). At follow-up in 2004–5, 2,346 participants kept a pedometer record, completed the International Physical Activity Questionnaire and/or a PWC_170_ fitness test; and had height and weight measured (overweight = BMI≥25 m/kg^2^).

**Results:**

At baseline and follow-up, median total physical activity, fitness and BMI were similar in participants who attended low, medium and high physical activity schools, and those attending high physical activity schools reported only modestly higher school physical activity. There was no difference in the prevalence of high total physical activity and fitness levels in childhood or adulthood across compulsory school physical activity categories. The prevalence of overweight in childhood and adulthood was similar across low, medium and high compulsory physical activity schools.

**Conclusion:**

The amount of compulsory physical activity reported by schools was not associated with total physical activity, fitness or overweight in childhood or in adulthood. Policies promoting amounts of compulsory school physical activity in this range may not be sufficient to increase physical activity and fitness or reduce the prevalence of obesity in children.

## Background

The benefits of regular participation in physical activity have been well-documented [1]. While monitoring and surveillance of children's physical activity has been limited, a recent review of secular trends in physical activity concluded that physical activity in clearly defined contexts, particularly school physical education (PE), active commuting and organised sport, has declined in many countries [2]. Coupled with rapidly increasing rates of overweight and obesity in children [3,4], these declines are cause for concern.

Schools have often been targeted as important settings for health promotion strategies aimed at increasing children's physical activity levels. In 2005 the Australian federal government introduced legislation requiring all primary and junior secondary schools to provide a minimum of two hours per week of physical activity [5]. This physical activity can be structured or unstructured but must be implemented through the curriculum during class times; it therefore seems likely that this compulsory physical activity will be undertaken through existing school PE and school sport programs. While structured interventions have had some success at increasing physical activity levels in the short [6,7] and longer term [8], the effectiveness of policy that mandates compulsory school physical activity as a strategy for increasing children's physical activity is unknown.

This study aimed to examine whether children who attended schools that reported providing higher levels of compulsory weekly physical activity (school PE and school sport) had higher physical activity and fitness levels and a lower prevalence of overweight than children whose schools reported providing lower levels of compulsory physical activity. A secondary aim was to examine whether reported compulsory school physical activity provision in childhood had any lasting effects on physical activity, cardiorespiratory fitness and overweight at follow-up 20 years later.

## Methods

### Participants

Data were derived from the 1985 Australian Schools Health and Fitness Survey (ASHFS), a nationally representative survey of 8,498 children aged 7–15 years from 109 primary and secondary government, Catholic and independent schools. Details of the sampling strategy have been described elsewhere [9,10]. Briefly, the first stage of sampling involved selecting schools with a probability proportional to the number of children aged 10 years in primary schools and 14 years in secondary schools (90.1% response rate). The second stage of sampling involved randomly selecting 10 children from each age and sex group (67.5% response rate).

During 2001–2, electoral rolls, electronic telephone directories, the National Death Index and contact with classmates were used to trace participants for the Childhood Determinants of Adult Health (CDAH) follow-up study [11]. Those contacted were invited to provide follow-up data and to attend one of 34 study clinics held around Australia for physical measurements during 2004–6. The CDAH study was approved by the Southern Tasmanian Medical Research Ethics Committee.

### Compulsory school physical activity

In 1985 senior school personnel, including principals, vice principals and sport coordinators, reported minutes of school physical education (PE) and school sport per timetable cycle, the number of days per timetable cycle and whether the activity was compulsory or voluntary. School PE aims to teach motor, fitness, personal and social skills, while school sport involves competition against oneself or another. Values for compulsory school PE and school sport were summed, divided by the number of days in the timetable cycle and multiplied by five to derive average weekly minutes of compulsory physical activity [11,12]. Average weekly minutes of compulsory school physical activity was then categorised into three groups – low, medium and high – based on tertile splits rounded to the nearest 10 minutes within school level strata (primary and secondary). Low, medium and high physical activity provision was defined as <110 minutes/week, 110–149 minutes per week and ≥150 minutes per week in primary schools; respective values for secondary schools were <150 minutes per week, 150–189 minutes/week and ≥190 minutes/week.

### Baseline outcome measures

Children aged 9–15 years (n = 6,412) completed a questionnaire in small groups with a trained data collector. This included past week duration and frequency of school physical activity (PE and sport) and non-school activity (walking and cycling to/from school and other exercise or sport). Body mass index (BMI) (kg/m^2^) was derived from measured height and weight (n = 6,554), with overweight and obesity defined using age-and sex-specific cutpoints [13]. Children aged 9, 12 and 15 years (n = 2,595) completed a bicycle ergometer test to estimate cardiorespiratory fitness from physical working capacity at a heart rate of 170 (PWC_170_). To account for lean body mass (LBM), PWC_170_ was divided by per cent body fat estimated from the sum of four skinfold thicknesses (W/kglbm) using the equations of Durnin and Rahaman [14]. Area-level socioeconomic status (SES) based on residential postcode was derived using the Australian Bureau of Statistics index of relative socioeconomic disadvantage from the 1981 population census [15]. Smoking, country of birth, parental smoking and parental physical activity were self-reported.

### Follow-up outcome measures

At follow-up, participants were aged 26–36 years (mean 31.0 ± 2.6). Data from pregnant women were excluded (n = 70). Participants were asked to wear a Yamax Digiwalker pedometer (model SW-200) for seven days; those participants who recorded daily steps for a minimum of eight hours per day for four days were included in this analysis (n = 1,786). 2,082 participants completed the long version of the International Physical Activity Questionnaire (IPAQ-L) [16]. Correlations between pedometer and IPAQ-L measures were *r *= 0.37 among men and *r *= 0.26 among women, consistent with findings from studies comparing self-reported and pedometer measures of physical activity [17]. BMI was derived from measured height and weight (n = 1,865), with BMI≥25 and <30 kg/m^2 ^classified as overweight and BMI≥30 kg/m^2 ^classified as obese. Cardiorespiratory fitness was estimated from a PWC_170_ bicycle ergometer test (n = 1,624) and was divided by per cent body fat estimated from the sum of four skinfold thicknesses (W/kglbm) to account for LBM, as at baseline. Highest level of education, current occupation, marital status and number of live births (females only) were self-reported.

### Analyses

Differences in the characteristics of participants and non-participants were examined using one-way analysis-of-variance (ANOVA) for normally distributed continuous variables, Kruskal-Wallis equality-of-populations rank tests when variances were not equal and chi-squared tests for categorical variables. These tests were conducted stratified by sex and school level.

Due to the skewed nature of the physical activity data, median and inter-quartile range (IQR) values are presented. Because of the non-normal distribution of the physical activity data, the Kruskal-Wallis equality-of-populations rank test was used to determine whether sex and school level (baseline only) differences existed; for consistency, the same test was used to examine sex and school level (baseline only) differences in cardiorespiratory fitness and BMI measures. Similarly, the Kruskal-Wallis test was used to determine whether significant differences existed in the physical activity, fitness and BMI levels of participants across the three categories (low, medium, high) of compulsory school physical activity provision.

Physical activity and cardiorespiratory fitness variables were dichotomised into two groups representing those in the top quarter ("high" physical activity or fitness) and those in all other quarters. Log binomial regression was used to calculate prevalence ratios (PR) and 95% confidence intervals (CIs) of high physical activity and high fitness, and overweight and obesity across the three categories of school physical activity provision (low, medium, high), adjusting standard errors for the effects of clustering by school using the Taylor-series approximation. Estimates are presented unadjusted and adjusted for sociodemographic factors that were significantly associated with the outcomes.

## Results

80.5% (n = 6,840) of the original sample were located, with 5,170 (60.8%) of those contacted agreeing to participate in the follow-up study. 2,410 (28.4%) of these attended a study clinic, with 2,390 (28.1%) providing data for these analyses. While there were some statistically significant baseline differences between those who participated in follow-up and those who did not, there were no significant differences in average baseline total, school or non-school physical activity, or in cardiorespiratory fitness levels. However, compared with non-participants, participants were more likely to have a higher area-level SES at baseline (primary school students only, 30% versus 25%), to have attended independent schools (primary school males only, 8% versus 4%), to have been born in Australia (secondary school females only, 95% versus 89%), to have spoken English at home (primary school females only, 90% versus 83%), and were less likely to be overweight (9% versus 13%). At follow-up the prevalence of smoking was less than in the general Australian population aged 25–34 years (20% current daily smokers versus 28–30%) [18]. The prevalence of overweight and obesity in the current study (64% of men and 40% of women) was similar to that found in 25–34 year olds in another large Australian population-based sample with objective measures of height and weight (61% of men and 35% of women) [19] and to that found in a national health survey using self-reported height and weight (61.5% of men and 37.1% of women) [20].

School PE was compulsory in 92.3% (n = 48/52) of primary schools and 98.1% (n = 52/53) of secondary schools, while school sport was compulsory in 86.4% (n = 38/44) of primary schools and 83.8% (n = 31/37) of secondary schools. 57.7% of primary schools and 81.5% of secondary schools provided at least two hours per week of compulsory physical activity. A description of compulsory physical activity provided by schools is given in Table 1.

**Table 1:** School physical activity provision in the 1985 Australian Schools Health and Fitness Survey

	**School Physical Activity Provision (mins/wk)**		
	
	**Low**	**Medium**	**High**
**Primary Schools**			
Median (IQR)	90 (75, 100)	120 (115, 140)	190 (165, 230)
Min., Max.	60, 105	110, 140	150, 340
**Secondary Schools**			
Median (IQR)	106 (88, 130)	170 (160, 180)	220 (200, 252)
Min., Max.	60, 146	150, 186	190, 773

Overall, there were few differences in median physical activity, fitness or BMI values or in the prevalence of overweight across categories of school physical activity provision at baseline (Table 2). However, there were significant differences in the amount of school-based physical activity reported by children at low, medium and high compulsory physical activity schools, with those children at high compulsory physical activity schools tending to report the most school-based physical activity. At follow-up, no differences were evident in the outcomes across school level strata therefore the school levels were combined. No significant differences were found in the median physical activity, fitness or BMI values or in the prevalence of overweight at follow-up across categories of compulsory school physical activity. Treating the dependent and independent variables continuously made little difference to the results. For instance, a 60-minute increase in the amount of school physical activity provided showed no significant association with children's total physical activity (boys: 2.3 minutes/week, 95% CI -6.7, 11.4; girls: 3.0 minutes/week, 95% CI -11.5, 5.4) or children's BMI (boys: -0.0008 kg/m^2^, 95% CI -0.0020, 0.0003; girls: -0.0002 kg/m^2^, 95% CI -0.0014, 0.0010), nor with adult total physical activity (men: 6.6 minutes/week, 95% CI -16.3, 29.4; women: -4.9 minutes/week, 95% CI -51.9, 42.0) or adult BMI (men: 0.002 kg/m^2^, 95% CI -0.001, 0.006; women: 0.002 kg/m^2^, 95% CI -0.03, 0.007).

**Table 2:** Physical activity, fitness & BMI at baseline and follow-up, by sex and physical activity provision

	**Males**			**Females**		
	
	**Low PA School**	**Medium PA School**	**High PA School**	**Low PA School**	**Medium PA School**	**High PA School**
**Child (Baseline)** **Measures**		**Median, IQR**			**Median, IQR**	
Total PA^a ^(mins/wk)						
Primary School	290 (150, 495)	285 (170, 490)	315 (177, 553)	243 (140, 435)	240 (140, 420)	260 (155, 450)
Secondary School	413 (230, 720)	470 (250, 720)	433 (240, 700)	350 (190, 570)	340 (200, 580)	335 (200, 550)
School PA^a ^(mins/wk)						
Primary School	90 (50, 130)	80 (50, 150)	100 (45, 150) ^b^	90 (60, 135)	90 (40, 140)	95 (45, 160)
Secondary School	120 (80, 200)	150 (90, 210)	150 (87, 203) ^b^	110 (50, 200)	150 (90, 200)	150 (89, 200) ^c^
Non-School PA^a ^(mins/wk)						
Primary School	2.9 (2.5, 3.3)	2.8 (2.3, 3.2)	3.0 (2.5, 3.4)	2.4 (2.1, 2.8)	2.3 (2.0, 2.7)	2.4 (2.1, 2.7)
Secondary School	3.2 (2.8, 3.7)	3.2 (2.7, 3.6)	3.1 (2.7, 3.5) ^c^	2.5 (1.9, 3.0)	2.6 (2.1, 3.0)	2.5 (2.2, 2.9)
BMI (kg/m^2^)						
Primary School	17.0 (15.9, 18.5)	17.0 (16.0, 18.7)	17.0 (15.9, 18.7)	17.3 (15.9, 19.0)	17.2 (15.9, 19.0)	17.2 (16.0, 19.0)
Secondary School	19.3 (17.7, 20.9)	19.3 (17.8, 21.1)	19.2 (17.7, 21.0)	20.0 (18.3, 21.6)	19.7 (18.1, 21.6)	19.6 (18.1, 21.5)
Overweight		**% (n)**			**% (n)**	
Primary School	7.6 (56)	6.6 (55)	6.7 (61)	7.4 (60)	8.3 (68)	7.9 (69)
Secondary School	9.5 (46)	11.3 (61)	10.7 (74)	11.8 (56)	12.5 (62)	12.1 (70)
**Adult (Follow-Up)** **Measures**		**Median, IQR**			**Median, IQR**	
Daily Steps	8730 (6840, 11360)	8731 (6558, 11005)	9021 (6830, 11459)	8827 (6882, 10723)	8519 (6715, 11036)	8311 (6425, 10758)
Total PA^a ^(mins/wk)	668 (375, 1070)	640 (360, 1097)	684 (375, 1160)	660 (360, 1060)	625 (365, 1080)	740 (380, 1123)
LTPA^a ^(mins/wk)	103 (0, 253)	96 (0, 219)	101 (0, 255)	90 (0, 219)	96 (0, 210)	95 (15, 240)
Fitness (W/kglbm)	3.0 (2.7, 3.4)	3.1 (2.7, 3.5)	3.0 (2.6, 3.5) b	2.8 (2.4, 3.3)	2.9 (2.6, 3.2)	2.9 (2.5, 3.3)
BMI (kg/m^2^)	26.0 (23.6, 28.6)	26.4 (23.9, 28.7)	26.2 (24.2, 28.6)	23.5 (21.5, 27.7)	23.6 (21.4, 27.4)	23.9 (21.7, 27.6)
		**% (n)**			**% (n)**	
Overweight	58.6 (187)	62.8 (245)	64.6 (263)	39.2 (136)	37.6 (136)	39.7 (156)

^a ^Self-reported physical activity (PA).

^b ^p < 0.05 from Kruskal-Wallis equality-of-populations rank test for differences across school PA provision categories.

^c ^p < 0.01 from Kruskal-Wallis equality-of-populations rank test for differences across school PA provision categories.

IQR: inter-quartile range; PA: physical activity; LTPA: leisure time physical activity; BMI: body mass index.

There was no association between the amount of compulsory school physical activity and the prevalence of being in the top quarter of physical activity or fitness (Table 3). Adjusting for area-level SES, smoking and parental physical activity made little difference to the estimates. Similarly, there was no association between compulsory school physical activity provision at baseline and the prevalence of being in the top quarter of physical activity or fitness at follow-up (Table 4). Adjustments for sociodemographic characteristics at baseline (area-level SES, smoking and parental physical activity) and follow-up (highest level of education, marital status, smoking status and in females only, number of live births) made little difference to the associations. There was also no association between school physical activity provision and the prevalence of overweight at baseline or at follow-up in males or in females (Table 5). Again, adjustment for sociodemographic characteristics made little difference to the estimates.

**Table 3:** Baseline associations between compulsory school physical activity & high levels of physical activity and fitness^a^

**Baseline Measures**	**Boys**					**Girls**				
	
	**% (n/N)**	**PR**^c^	**95% CI**	**PR**^d^	**95% CI**	**% (n/N)**	**PR**^c^	**95% CI**	**PR**^d^	**95% CI**
Total PA^b^										
Low PA School	25 (205/812)	1.0	Ref	1.0	Ref	27 (229/860)	1.0	Ref	1.0	Ref
Med PA School	25 (234/945)	0.98	(0.81–1.19)	0.98	(0.80–1.19)	25 (228/911)	0.94	(0.75–1.18)	0.93	(0.75–1.15)
High PA School	26 (299/1128)	1.05	(0.86–1.28)	1.05	(0.85–1.28)	26 (262/1004)	0.98	(0.79–1.21)	0.95	(0.77–1.16)
*p*_*trend*_			0.60		0.67			0.87		0.70
School PA^b^										
Low PA School	23 (183/812)	1.0	Ref	1.0	Ref	40 (341/860)	1.0	Ref	1.0	Ref
Med PA School	27 (256/945)	1.20	(0.90–1.60)	1.20	(0.91–1.58)	38 (348/911)	0.96	(0.73–1.27)	0.95	(0.74–1.21)
High PA School	28 (316/1128)	1.24	(0.91–1.70)	1.24	(0.91–1.68)	42 (417/1004)	1.05	(0.78–1.41)	1.02	(0.78–1.33)
*p*_*trend*_			0.19		0.20			0.75		0.87
Non-School PA^b^										
Low PA School	25 (200/812)	1.0	Ref	1.0	Ref	27 (232/860)	1.0	Ref	1.0	Ref
Med PA School	26 (244/945)	1.05	(0.88–1.25)	1.04	(0.87–1.25)	26 (238/911)	0.97	(0.81–1.16)	0.98	(0.82–1.16)
High PA School	27 (308/1128)	1.11	(0.92–1.34)	1.11	(0.91–1.34)	26 (256/1004)	0.95	(0.78–1.15)	0.93	(0.77–1.13)
*p*_*trend*_			0.28		0.30			0.58		0.51
Fitness										
Low PA School	28 (90/323)	1.0	Ref	1.0	Ref	30 (97/326)	1.0	Ref	1.0	Ref
Med PA School	22 (83/382)	0.78	(0.56–1.09)	0.78	(0.56–1.09)	26 (94/367)	0.86	(0.59–1.25)	0.86	(0.61–1.23)
High PA School	25 (111/453)	0.88	(0.63–1.22)	0.88	(0.63–1.22)	25 (109/433)	0.85	(0.62–1.15)	0.85	(0.64–1.13)
*p*_*trend*_			0.52		0.53			0.30		0.27

^a ^High physical activity and fitness defined as being in the top quarter of physical activity or fitness within sex and school level strata.

^b ^Self-reported physical activity (PA).

^c ^PR: prevalence ratios adjusted for the effects of clustering by school.

^d ^PR: prevalence ratios adjusted for the effects of clustering by school plus area-level SES, smoking and parental physical activity.

**Table 4:** Follow-up associations between school physical activity provision and high levels of physical activity and fitness^a^

**Follow-Up Measures**	**Men**					**Women**				
	
	**% (n/N)**	**PR**^c^	**95% CI**	**PR**^d^	**95% CI**	**% (n/N)**	**PR**^c^	**95% CI**	**PR**^d^	**95% CI**
Daily Steps										
Low PA School	27 (55/205)	1.0	Ref	1.0	Ref	26 (68/263)	1.0	Ref	1.0	Ref
Med PA School	23 (53/235)	0.84	(0.59–1.20)	0.91	(0.65–1.26)	27 (71/267)	1.03	(0.73–1.45)	1.00	(0.75–1.34)
High PA School	28 (77/278)	1.03	(0.74–1.44)	1.09	(0.79–1.50)	28 (80/288)	1.07	(0.76–1.52)	1.05	(0.77–1.43)
*p*_*trend*_			0.77		0.48			0.68		0.81
Total PA^b^										
Low PA School	22 (52/237)	1.0	Ref	1.0	Ref	27 (86/321)	1.0	Ref	1.0	Ref
Med PA School	23 (62/266)	1.06	(0.76–1.49)	1.08	(0.79–1.47)	26 (87/336)	0.97	(0.72–1.29)	0.95	(0.74–1.24)
High PA School	26 (79/305)	1.18	(0.84–1.66)	1.22	(0.90–1.66)	28 (100/357)	1.05	(0.82–1.34)	1.00	(0.78–1.28)
*p*_*trend*_			0.32		0.19			0.71		0.99
LTPA^b^										
Low PA School	30 (70/237)	1.0	Ref	1.0	Ref	23 (73/321)	1.0	Ref	1.0	Ref
Med PA School	23 (61/266)	0.78	(0.59–1.03)	0.79	(0.60–1.04)	24 (80/336)	1.05	(0.80–1.36)	1.10	(0.84–1.44)
High PA School	27 (83/305)	0.92	(0.70–1.22)	0.92	(0.71–1.20)	26 (93/357)	1.15	(0.89–1.47)	1.18	(0.91–1.53)
*p*_*trend*_			0.65		0.57			0.29		0.22
Fitness										
Low PA School	23 (43/190)	1.0	Ref	1.0	Ref	26 (51/197)	1.0	Ref	1.0	Ref
Med PA School	30 (68/229)	1.31	(0.90–1.92)	1.41	(1.00–1.97)	22 (46/209)	0.85	(0.58–1.25)	0.82	(0.58–1.17)
High PA School	23 (62/266)	1.03	(0.71–1.48)	1.14	(0.81–1.60)	26 (61/235)	1.00	(0.70–1.43)	0.98	(0.72–1.35)
*p*_*trend*_			0.98		0.75			0.95		0.90

^a ^High physical activity and fitness defined as being in the top quarter of physical activity or fitness within sex strata.

^b ^Self-reported physical activity (PA).

^c ^PR: prevalence ratios adjusted for the effects of clustering by school.

^d ^PR: prevalence ratios adjusted for the effects of clustering by school plus baseline area-level SES, baseline smoking, baseline parental physical activity, highest level of education, smoking at follow-up, marital status at follow-up and number of live births at follow-up (females only)

**Table 5:** Associations between school physical activity provision and prevalence of overweight^a ^at baseline and follow-up

	**Males**					**Females**				
	
	**% (n/N)**	**PR**^b^	**95% CI**	**PR**^c^	**95% CI**	**% (n/N)**	**PR**^c^	**95% CI**	**PR**^b^	**95% CI**
**Baseline**										
*Overweight/Obese*										
Primary School										
Low PA School	14 (51/371)	1.0	Ref	1.0	Ref	12 (48/417)	1.0	Ref	1.0	Ref
Med PA School	10 (48/460)	0.76	(0.52–1.11)	0.80	(0.57–1.12)	13 (60/455)	1.15	(0.81–1.61)	1.14	(0.80–1.62)
High PA School	11 (53/503)	0.77	(0.55–1.06)	0.77	(0.56–1.07)	13 (59/472)	1.09	(0.80–1.48)	1.10	(0.83–1.47)
*p*_*trend*_			0.13		0.14			0.62		0.51
Secondary School										
Low PA School	9 (40/440)	1.0	Ref	1.0	Ref	12 (51/442)	1.0	Ref	1.0	Ref
Med PA School	11 (55/485)	1.25	(0.78–1.99)	1.27	(0.86–1.88)	13 (57/456)	1.08	(0.72–1.63)	1.15	(0.80–1.65)
High PA School	11 (68/622)	1.20	(0.82–1.76)	1.14	(0.79–1.64)	13 (69/532)	1.12	(0.80–1.59)	1.15	(0.81–1.64)
*p*_*trend*_			0.39		0.59			0.50		0.44
**Follow-Up**										
*Overweight/Obese*										
Low PA School	59 (123/208)	1.0	Ref	1.0	Ref	39 (92/239)	1.0	Ref	1.0	Ref
Med PA School	66 (169/254)	1.13	(0.97–1.30)	1.10	(0.97–1.25)	38 (96/250)	1.00	(0.78–1.28)	0.98	(0.79–1.22)
High PA School	63 (182/288)	1.07	(0.93–1.23)	1.08	(0.95–1.23)	41 (110/270)	1.06	(0.83–1.34)	1.04	(0.84–1.28)
*p*_*trend*_			0.46		0.33			0.63		0.63

^a ^Overweight at baseline defined as a BMI equal to or greater than international age- and sex-specific cutpoints for overweight; overweight at follow-up defined as a BMI≥25 kg/m^2^.

^b ^PR: prevalence ratios adjusted for the effects of clustering by school.

^c ^PR: prevalence ratios adjusted for the effects of clustering by school plus baseline area-level SES, baseline smoking, baseline parental physical activity; follow-up associations also adjusted for highest level of education, smoking at follow-up, marital status at follow-up and number of live births at follow-up (females only).

## Discussion

School physical activity has a range of intended benefits including improved motor skills, fitness, social interaction and learning [12]. Policies that make school physical activity compulsory are also seen as an opportunity for health promotion through increasing children's overall energy expenditure and thereby reducing the risk of obesity. This study aimed to examine whether children who attended schools that reported higher levels of compulsory physical activity had higher levels of physical activity and cardiorespiratory fitness and a lower prevalence of overweight than children who attended schools reporting lower levels of compulsory physical activity. Overall, the findings suggest that the amount of compulsory school physical activity reported had little effect on children's total physical activity and fitness and on the prevalence of overweight and obesity. However, children at schools providing more compulsory school physical activity tended to report more school-based physical activity than children at schools providing less compulsory physical activity.

Our cross-sectional findings for children are consistent with some previous research. A study of children (n = 215) at three schools in England found that irrespective of the time spent in physical activity at school (9, 2.2 or 1.8 hours per week), there was no difference in children's total physical activity as measured by accelerometer [21]. Similarly, a two-year Nebraskan trial found that children (n = 338 at baseline) who received enhanced physical activity (30–40 minutes 3 days per week), grade specific nutrition education and a modified school lunch program had classroom physical activity levels 6% higher than controls, but outside of school physical activity was 16% lower, and no significant differences in body mass index (BMI) were noted in a subsample (n = 108) [22]. In contrast, a South Australian school-based physical activity trial observed increases in children's fitness (n = 513) and decreases in body fat (n = 510) following an intervention that included 6.25 hours (375 minutes) of high intensity, structured physical activity per week [23]. In the current study, schools provided a median of 2.67 hours (160 minutes) per week. This suggests that larger amounts of school-based physical activity conducted at high intensities under strict intervention conditions may be required to see differences in total physical activity, fitness and body mass.

Attending a school that reported higher levels of compulsory physical activity was not associated with adult physical activity, cardiorespiratory fitness or weight status. To our knowledge, this is the first study to investigate the long-term effects of compulsory school physical activity under non-intervention conditions. One Canadian study followed up 147 children 20 years after they received a specialist-taught PE intervention from grades 1 through 6 [24]. Similar to the current study, no significant differences in adult adiposity in either sex or physical activity levels in males were observed, although a higher proportion of females in the experimental group (35.5%) reported participating in strenuous physical activity three or more times per week, compared with the comparison group (20.2%) [25]. Our findings are consistent with the generally low levels of tracking observed between child and adult physical activity (*r *= 0.05–0.54) [26]. While it is of public health interest to examine the long-term impacts of childhood exposures (such as exposure to higher school physical activity provision), there are a large number of external influences on physical activity behaviors during childhood, youth and young adulthood [27,28] that may override any effects of school physical activity provision.

Our study had some limitations. Children's self-reported physical activity is prone to measurement error [29] which may bias findings towards the null. However, this seems unlikely to have substantially affected our results because objectively measured cardiorespiratory fitness, which closely reflects an individual's physical activity levels, demonstrated similar associations. Also, children's self-reported physical activity was reported at levels consistent with those observed in other studies [30,31] and was correlated with cardiorespiratory fitness at similar levels to that observed previously (Spearman's *rho *= 0.17) [32,33]. While measurement of adult levels of physical activity can also be problematic, we attempted to overcome this by using both self-reported and objective measures, which have been shown to be validly and reliably used in large population-based studies [16,17,34-37]. Average values for self-reported total physical activity were similar to those reported in other studies using the IPAQ-L [16], and average daily steps were similar to those observed in an Australian study of 18–29 year olds [38], although studies in other populations have demonstrated lower [39] and higher [40] average daily step values. These measures however do suffer from limitations; the IPAQ-L has been criticized for overestimating physical activity [41], while pedometers are unable to capture non-ambulatory activities such as swimming, cycling or weight-training. School-reported physical activity and child-reported physical activity were not well-correlated; school reports of compulsory physical activity provision may also have been prone to measurement error. This could have resulted, for example, from different amounts of school physical activity being provided to different grades or from the effects of social desirability prompting schools to over-report their school physical activity.

A limitation of the follow-up analyses is that only 28% of the original sample had follow-up measurements. While there were some small sociodemographic differences at baseline (as described in the Results), there were no significant differences in physical activity or cardiorespiratory fitness values at baseline between those who participated in follow-up and those who did not. The prevalence of overweight at baseline was lower in those who participated in follow-up although the adult sample had similar levels of overweight and obesity to Australians of the same age [19,20]. Whether an association between school physical activity provision in childhood and adult physical activity, fitness and overweight is different in those who did not participate is unknown.

A range of unmeasured factors such as school resources, interest and parental expectations may have contributed to schools' compulsory physical activity policies in 1985. These could be potential confounders in the association between school physical activity provision and our outcome measures of physical activity, fitness and overweight. Also, the degree to which findings from 1985 translate to present day school environments is not clear. Nevertheless, given that no other national dataset of this nature and size currently exists in Australia, and no previous studies have examined long-term influences of compulsory school physical activity on adult physical activity, fitness and overweight, this study is uniquely placed to contribute to debate about the likely benefits of compulsory school physical activity. This study also has key strengths in its size, length of follow-up, extensive range of behavioral and biological measures and ability to adjust for a range of potentially confounding factors.

While compulsory school physical activity may bring a range of physical and social benefits to children [42] our findings suggest that policies promoting or requiring compulsory physical activity of up to 190 minutes per week in primary schools and 220 minutes per week in secondary schools may be insufficient to increase total physical activity and fitness levels or decrease the prevalence of overweight in childhood or in the longer term.

## Competing interests

The authors declare that they have no competing interests.

## Authors' contributions

VC carried out the analyses and drafted the manuscript. AV and TD participated in study conceptualisation, design and coordination and helped to draft the manuscript. LB provided statistical advice and input into conceptualisation of the paper. All authors read and approved the final manuscript.
